# Association between canine leishmaniosis and *Ehrlichia canis* co-infection: a prospective case-control study

**DOI:** 10.1186/s13071-018-2717-8

**Published:** 2018-03-20

**Authors:** Charalampos Attipa, Laia Solano-Gallego, Kostas Papasouliotis, Francesca Soutter, David Morris, Chris Helps, Scott Carver, Séverine Tasker

**Affiliations:** 1 Molecular Diagnostic Unit, Diagnostic Laboratories, Bristol Veterinary School and Langford Vets, University of Bristol, Langford, UK; 2 Department of Pathobiology and Population Sciences, The Royal Veterinary College, University of London, Hatfield, Hertfordshire, UK; 3 Cyvets Veterinary Center, Paphos, Cyprus; 4 Departament de Medicina i Cirurgia Animals, Facultat de Veterinària, Universitat Autònoma de Barcelona, Barcelona, Spain; 5 Department of Biological Sciences, University of Tasmania, Tasmania, Australia; 6 Bristol Veterinary School, University of Bristol, Langford, UK; 7 IDEXX Laboratories Ltd, Wetherby, UK

**Keywords:** Canine leishmaniosis, *Leishmania infantum*, *Ehrlichia canis*, Vector-borne pathogen, Co-infection, Cyprus, *Anaplasma platys*, *Mycoplasma haemocanis*, *Hepatozoon* spp., Structural equation model

## Abstract

**Background:**

In the Mediterranean basin, *Leishmania infantum* is a major cause of disease in dogs, which are frequently co-infected with other vector-borne pathogens (VBP). However, the associations between dogs with clinical leishmaniosis (ClinL) and VBP co-infections have not been studied. We assessed the risk of VBP infections in dogs with ClinL and healthy controls.

**Methods:**

We conducted a prospective case-control study of dogs with ClinL (positive qPCR and ELISA antibody for *L*. *infantum* on peripheral blood) and clinically healthy, ideally breed-, sex- and age-matched, control dogs (negative qPCR and ELISA antibody for *L*. *infantum* on peripheral blood) from Paphos, Cyprus. We obtained demographic data and all dogs underwent PCR on EDTA-blood extracted DNA for haemoplasma species, *Ehrlichia*/*Anaplasma* spp., *Babesia* spp., and *Hepatozoon* spp., with DNA sequencing to identify infecting species. We used logistic regression analysis and structural equation modelling (SEM) to evaluate the risk of VBP infections between ClinL cases and controls.

**Results:**

From the 50 enrolled dogs with ClinL, DNA was detected in 24 (48%) for *Hepatozoon* spp., 14 (28%) for *Mycoplasma haemocanis*, 6 (12%) for *Ehrlichia canis* and 2 (4%) for *Anaplasma platys*. In the 92 enrolled control dogs, DNA was detected in 41 (45%) for *Hepatozoon* spp., 18 (20%) for *M*. *haemocanis*, 1 (1%) for *E. canis* and 3 (3%) for *A*. *platys*. No *Babesia* spp. or “*Candidatus* Mycoplasma haematoparvum” DNA was detected in any dog. No statistical differences were found between the ClinL and controls regarding age, sex, breed, lifestyle and use of ectoparasitic prevention. A significant association between ClinL and *E. canis* infection (OR = 12.4, 95% CI: 1.5–106.0, *P* = 0.022) was found compared to controls by multivariate logistic regression. This association was confirmed using SEM, which further identified that younger dogs were more likely to be infected with each of *Hepatozoon* spp. and *M*. *haemocanis*, and dogs with *Hepatozoon* spp. were more likely to be co-infected with *M*. *haemocanis*.

**Conclusions:**

Dogs with ClinL are at a higher risk of co-infection with *E. canis* than clinically healthy dogs. We recommend that dogs diagnosed with ClinL should be tested for *E. canis* co-infection using PCR.

## Background

Canine leishmaniosis, caused by the protozoan parasite *Leishmania infantum,* is transmitted by a phlebotomine sand fly vector [1] and is endemic in Central and South America, Asia and several countries of the Mediterranean basin. An estimated 2.5 million dogs are infected with *L. infantum* in south-west Europe alone [2]. This potentially fatal protozoal infection of dogs and humans is an ideal example of the “One Health” approach to disease since dogs are the major reservoir of infection for humans [3]. In addition, an increasing number of canine leishmaniosis cases are being reported in non-endemic European countries, such as the UK and Germany, due to pet travel and importation of dogs from endemic areas, making leishmaniosis an emerging disease in these countries [4–6]. There is a risk that it might become endemic in such countries if future climate conditions support the life-cycle of a suitable vector.

Dogs with clinical leishmaniosis (ClinL) are often concurrently infected with multiple pathogens, which are often vector-borne, such as *Ehrlichia canis*, the causative agent for canine monocytic ehrlichiosis, *Anaplasma platys*, *Babesia vogeli* and *Hepatozoon canis*, resulting in an unpredictable incubation period, atypical clinical outcome and poorer prognosis, compared with dogs infected with *L. infantum* alone [7, 8]. These vector-borne pathogens (VBP) are transmitted by different vectors to dogs*,* such as *Rhipicephalus sanguineus* (for *A*. *platys*, *E. canis* and *H*. *canis*), *Ixodes ricinus* (for *Anaplasma phagocytophilum*), *Ixodes* spp. ticks (for *Borrelia burgdorferi*) and mosquitoes (for *Dirofilaria immitis*) [9]. While it has been suggested that leishmaniosis is a predisposing factor for infection with other pathogens in dogs, this has not been investigated to date [8, 10].

The aim of this case-control study was to investigate the hypothesis that dogs with ClinL are at greater risk for VBP infections than clinically healthy dogs. In addition, besides the commonly used logistic regression analyses for case-control studies [11], we performed structural equation modelling (SEM), which is an advancement of traditional regression approaches, allowing direct, indirect and co-variance relationships to be assessed simultaneously. The SEM has recently been employed in veterinary studies [12].

## Methods

### Study design and populations

Through a case-control study design, we evaluated if dogs with ClinL are at a greater risk than healthy controls for VBP infections including *Babesia* spp., “*Candidatus* Mycoplasma haematoparvum” (*CMhp*)*, Ehrlichia/Anaplasma* spp., *Hepatozoon* spp., and *M. haemocanis*. All dogs presented as clinical patients to a veterinary centre in Paphos, Cyprus, an area with high prevalence of *L*. *infantum* in dogs [13] and endemic for canine VBPs [14].

Eligible cases included dogs naturally infected with ClinL which were diagnosed based on the presence of clinical signs associated with *L*. *infantum* infection, and enrolled in the final statistical analysis if they were positive on both quantitative PCR (qPCR) on peripheral blood and serum antibodies for *L*. *infantum*. We attempted to match controls to the cases by age, sex and breed as well as, if possible, by lifestyle and the use of ectoparasitic prevention. For ClinL crossbreed dogs, the controls were dogs of similar size and dog group (e.g. terrier, toy or hound group) to the case dog. The control dogs were apparently clinically healthy, and were enrolled in the final statistical analysis if they were negative by both qPCR and antibody serology for *L*. *infantum* on peripheral blood.

Data on age, sex (male or female), breed (pedigree or crossbreed), lifestyle (outdoors or mainly indoors), use of ectoparasitic prevention (use or no use) and clinical signs were recorded for each dog. All dogs were examined by the same veterinarian author (CA) and classified as clinically healthy or suffering from ClinL, following The LeishVet Group Guidelines [15]. Exclusion criteria for enrolment in this study included prior vaccination or treatment for leishmaniosis, dogs undergoing therapy with immunosuppressives/chemotherapeutics or dogs less than 6 months old.

### Laboratory tests

We obtained blood samples of approximately 2–4 ml in plain and EDTA blood tubes by venepuncture from each dog. The EDTA blood tubes were centrifuged; plasma samples were obtained and transferred in a separate tube. All tubes were frozen at -20 °C until transported on dry ice to the Department of Pathobiology and Population Sciences, The Royal Veterinary College, University of London, Hatfield, Hertfordshire, UK. For the PCRs, DNA was extracted from 200 μl of EDTA blood using a commercial kit GenEluteTM Blood Genomic DNA Kit (Sigma-Aldrich, Dorset, UK) according to the manufacturer’s instructions. During extraction, nuclease-free water was used as a negative extraction control. The DNA was eluted with 50 μl of nuclease free water and stored at -20 °C until transported on dry ice to Diagnostic Laboratories, Langford Vets, University of Bristol, UK, for testing.

In order to assess the presence of amplifiable DNA, the absence of PCR inhibitors and correct assay setup, the qPCRs for *Leishmania* spp. [16], *Babesia* spp. [17], *CMhp* and *M. haemocanis* [18] were duplexed with an internal amplification control (glyceraldehyde-3-phosphate dehydrogenase gene), and a threshold cycle (Ct) value of < 30 was used as a cut-off for indication of acceptable DNA. Any samples with Ct values greater than or equal to 30 were excluded from the study due to insufficient quantity/quality of DNA. Conventional PCR assays, as previously described, were used to detect infection with *Ehrlichia/Anaplasma* spp. [19] and *Hepatozoon* spp. [20]. For each PCR assay, DNA from known infected dogs and nuclease-free water were used as positive and negative controls, respectively.

All samples that yielded positive results with the *Ehrlichia/Anaplasma* spp. PCR assay and 1/3 of the positive *Hepatozoon* spp. samples (a mixture of ClinL cases and controls) were purified using the NucleoSpin PCR and Gel Clean-up kit (Macherey-Nagel, Düren, Germany) according to the manufacturer’s instructions, quantified with a Qubit™ fluorometer (Thermo Fisher Scientific, Paisley, UK) and submitted for DNA sequencing at DNA Sequencing and Services (College of Life Sciences, University of Dundee, Scotland), in both directions using the same primers as those used for the PCR. The forward and reverse DNA sequences were then assembled, and a consensus sequence was searched against the NCBI database using BLAST (www.ncbi.nlm.nih.gov/BLAST) to identify the infecting species.

For the *L*. *infantum* serology, sera from cases and controls were transported on dry ice to the Departament de Medicina i Cirurgia Animals, Facultat de Veterinària, Universitat Autònoma de Barcelona, Barcelona, Spain. A *L*. *infantum* enzyme-linked immunosorbent assay (ELISA), as previously described, was used [21]. Each ELISA also included a calibrator serum sample from a dog infected by *L. infantum* as confirmed by IFAT (IFI Megascreen FLUOLEISH inf, Diagnostik Megacor, Hörbranz, Austria), a commercially available ELISA (Esteve Veterinary Laboratories, Dr Esteva S.A, Barcelona, Spain) and a rapid immunomigratory test (Speedleish, Virbac, La Seyne sur Mer, France). The ELISA also included a positive control serum sample from a dog with confirmed *L. infantum* infection by IFAT and demonstrating clinical signs associated with *Leishmania* infection, as well as a negative control serum sample from a cat that was resident in the UK where *L. infantum* is not endemic. Results were quantified as ELISA units (EU) relative to the calibrator (arbitrarily set at 100 EU). The positive cut-off value had previously been established at 35 EU (mean + 4 standard deviations of values from 80 dogs from a non-endemic area).

### Statistical analysis

We calculated the sample size to allow the identification of risk for VBP co-infection in dogs with ClinL as follows. On the basis of the admission frequencies for VBPs in the study’s veterinary centre and previously published data [14, 22–24] the expected proportion of control dogs being exposed to VBPs was estimated at 5%. The power calculation was performed using the on-line EpiTools epidemiological calculator (http://epitools.ausvet.com.au). A sample size of 50 dogs with ClinL and 50 controls was calculated, when the testing hypothesis was set with an odds ratio of 6, a power of 80% and confidence level at 95%. To strengthen the statistical power, we used approximately a 1:2 ratio for matching. We compared the continuous variable (age) between ClinL cases and controls with the Mann-Whitney test and categorical variables (sex, breed, lifestyle, use of ectoparasitic prevention, positivity for *A*. *platys*, positivity for *E. canis*, positivity for *Hepatozoon* spp. and positivity for *M*. *haemocanis*) with the Chi-square test. Independent variables that yielded *P-*values of < 0.1 in a univariable analysis were then tested in a multivariable logistic regression analysis. Within the final multivariable models a *P-*value ≤0.05 was considered statistically significant for inclusion. Descriptive statistics and multivariable logistic regression analysis was carried out using SPSS for Windows (version 22.0; SPSS Inc., Chicago, IL, USA).

We constructed a SEM that reflected the two hypothesised mechanisms associated with ClinL and VBPs infection statuses in domestic dogs: (i) causal effects of host characteristics; and (ii) pathogen interrelationships. We modelled the host characteristics as variables that predicted VBPs status, except ClinL which was controlled for in the sampling design. To estimate VBP interrelationships, including potential pathogen-facilitation, we included pathogen-pathogen covariance in the model. We followed Kline [25] and Rosseel [26], and more recent package advancements available through the R package *lavaan* (www.lavaan.ugent.be) to check alignment with SEM assumptions. Model fit was assessed using a chi-square statistic, and additionally scrutinized using a root mean square error of approximation and a comparative fit index, as recommended by Kline [25]. We used a diagonally weighted least squares SEM estimator method, which is appropriate for endogenous categorical variables [25, 26]. We present standardised coefficients and covariances enabling comparison among coefficient effect sizes [25, 26]. All SEM analyses were undertaken in the program R version 3.1.2 (www.r-project.org) using the *lavaan* [26] package.

## Results

From March 2013 to April 2014, 53 dogs with ClinL and 103 dog controls were screened for eligibility. We excluded three dogs with ClinL; two were ELISA-positive but qPCR-negative and one was qPCR-positive but ELISA-negative for *L*. *infantum*. From the controls dogs 11 were excluded; nine were qPCR-positive and two were ELISA-positive for *L*. *infantum*. The age of the 142 dogs enrolled in the case-control study ranged from 1 to 12 years (median 5.6 years, interquartile range 8 years) and 105 (74%) were pedigree. The most common breeds were Segugio Italiano, Cocker Spaniel, German Shepherd, Beagle and German Shorthair Pointer.

**Fig. 1:**
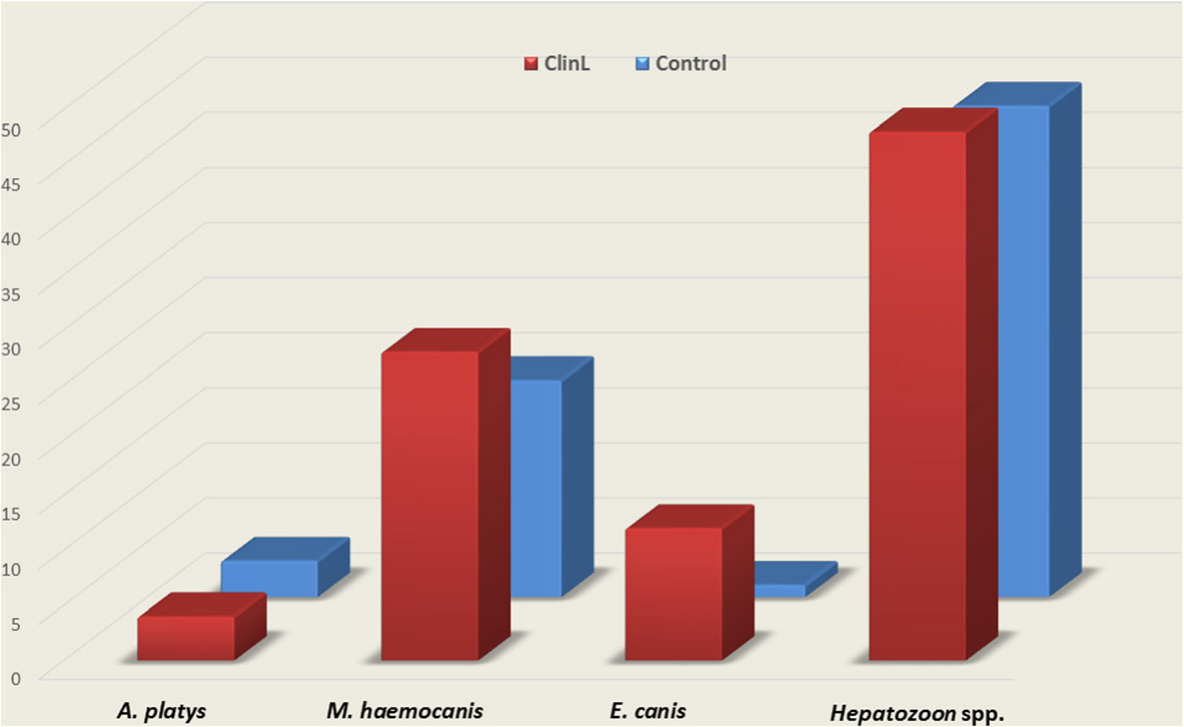
Comparison of VBPs detected by PCR and sequencing between dogs with ClinL (*n* = 50) and healthy control (*n* = 92). *Abbreviations*: VBP, vector-borne pathogen; ClinL, clinical leishmaniosis; *A*. *platys*, *Anaplasma platys*; *E. canis, Ehrlichia cani*; *M*. *haemocanis*, *Mycoplasma haemocanis*

From the 50 enrolled dogs with ClinL, DNA was detected in 24 (48%) for *Hepatozoon* spp., 14 (28%) for *M. haemocanis*, 6 (12%) for *E. canis* and 2 (4%) for *A. platys*. In the 92 enrolled control dogs, DNA was detected in 41 (45%) for *Hepatozoon* spp., 18 (20%) for *M*. *haemocanis*, 1 (1%) for *E. canis* and 3 (3%) for *A*. *platys* (Fig. 1). Only *H*. *canis* was identified following sequencing of *Hepatozoon* spp. PCR-positive samples. No *Babesia* spp. or “*Candidatus* Mycoplasma haematoparvum” DNA was detected in any dog. Table 1 summarizes the characteristics and the PCR results for the VBPs tested.

**Table 1:** Characteristics of the study dog groups and PCR/sequencing results for the VBPs tested. All dogs tested negative on quantitative PCR for *Babesia* spp. and “*Candidatus* Mycoplasma haematoparvum”. The species of *A*. *platys* and *E. canis* were identified following sequencing of PCR products derived from generic *Ehrlichia*/*Anaplasma* PCR testing

Characteristic	No. of cases ClinL (%) (*n* = 50)	Control (%) (*n* = 92)
Age in years		
Median	3	4
Interquartile range	3.3	3.0
Sex		
Male	24 (48)	50 (54)
Female	26 (52)	42 (46)
Lifestyle		
Outoors	35 (70)	68 (74)
Mainly indoors	15 (30)	24 (26)
Ectoparasitic prevention		
Used	17 (34)	38 (41)
Not used	33 (66)	54 (69)
Breed		
Pedigree	35 (70)	70 (76)
Crossbreed	15 (30)	22 (24)
*A*. *platys*		
Positive	2 (4)	3 (3)
Negative	48 (96)	89 (97)
*E. canis*		
Positive	6 (12)	1 (1)
Negative	44 (88)	91 (99)
*Hepatozoon* spp.		
Positive	24 (48)	41 (45)
Negative	26 (52)	51 (55)
*M*. *haemocanis*		
Positive	14 (28)	18 (20)
Negative	36 (72)	74 (80)

*Abbreviations*: VBP, vector-borne pathogen; ClinL, clinical leishmaniosis; *A*. *platys*, *Anaplasma platys*; *E*. *canis*, *Ehrlichia canis*; *M*. *haemocanis*, *Mycoplasma haemocanis*

Using multivariable logistic regression analysis, a significant association between ClinL and *E. canis* infection [odds ratio = 12.4, 95% confidence interval (CI): 1.5–106.0, *P* = 0.022] compared to control dogs was found. We did not identify any association for *A*. *platys*, *Hepatozoon* spp. and *M*. *haemocanis* between the two groups. There were no statistically significant differences between the ClinL cases and controls in terms of age, sex, breed, lifestyle, and use of ectoparasitic prevention.

**Fig. 2:**
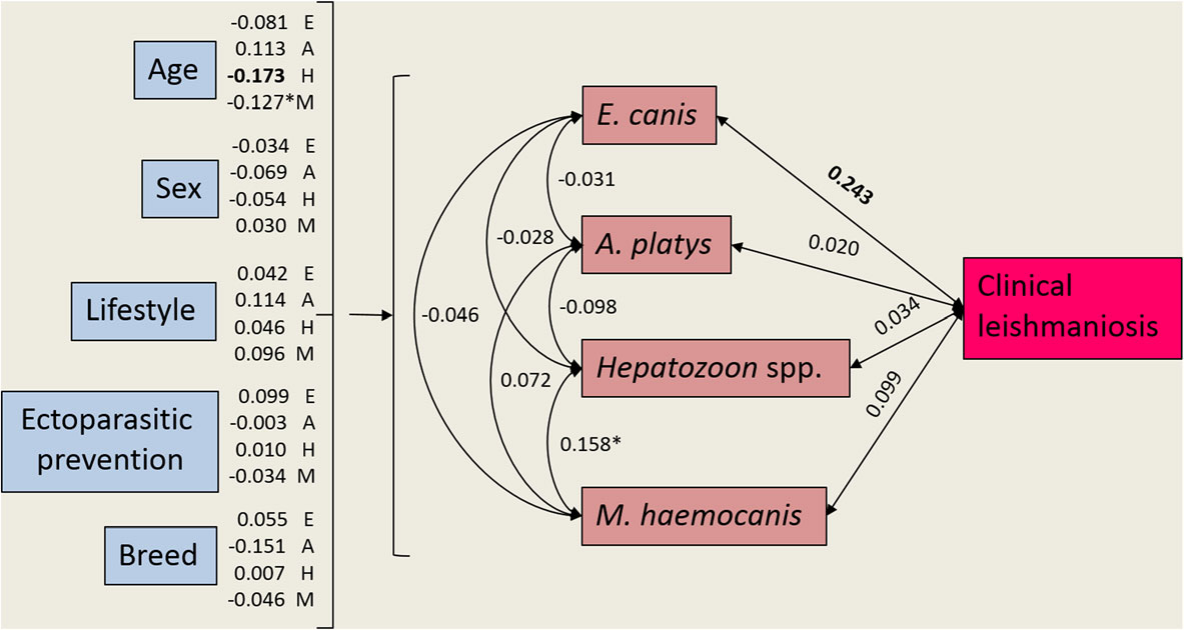
Structural equation model showing predictors of vector-borne co-infection (except *Leishmania infantum*), and pathogen covariance (including *L. infantum*). Values represent standardised coefficients among variables. Single headed arrows represent directional/causal relationships and double headed arrows covariance relationships among pathogens. For image clarity the coefficients of host characteristics predicting pathogens are listed next to each host characteristic. In all cases, except age, variables are binomial (0 or 1) with 1 equal to male, outside, ectoparasitic prevention use, pedigree and positive pathogen status. Significant relationships (*P* ≤ 0.05) denoted by bold font and trending relationships (*P* < 0.1) denoted by *. *Abbreviations*: *A*. *platys*, *Anaplasma platys*; *E. canis*, *Ehrlichia canis*; *M*. *haemocanis*, *Mycoplasma haemocanis.* *Note*: Values represent standardised coefficients among variables. Single headed arrows represent directional/causal relationships and double headed arrows covariance relationships among pathogens. For image clarity the coefficients of host characteristics predicting pathogens are listed next to each host characteristic. In all cases, except age, variables are binomial (0 or 1) with 1 equal to male, outside, ectoparasitic prevention use, pedigree and positive pathogen status. Significant relationships (*P* ≤ 0.05) denoted by bold font and trending relationships (*P* < 0.1) denoted by *.

The SEM supported four main associations among variables (Fig. 2, Table 2). Dogs with ClinL were more likely to be co-infected with *E. canis*, younger dogs were more likely to be infected with each of *Hepatozoon* spp. and *M*. *haemocanis,* although only a trend was identified for the latter, and a trend existed for co-infections between *Hepatozoon* spp. and *M*. *haemocanis* to occur. The SEM showed that there was otherwise negligible evidence of determinants of, or correlations among, VBPs.

**Table 2:** Structural equation model statistical output showing host characteristics predicting infection status for co-infecting pathogens (except *Leishmania infantum*), and the covariance among pathogens (including *L. infantum*), in domestic dogs. In all cases, except age, variables are binomial (0 or 1) with 1 equal to male, outside, ectoparasitic prevention use, pedigree and positive pathogen status

	Standardised coefficient/covariance	*z*-value	*P*-value
*E. canis*			
Age	-0.081	-0.790	0.429
Sex	-0.034	-0.391	0.696
Lifestyle	0.042	0.351	0.726
Ectoparasite prevention	0.099	0.749	0.454
Breed	0.055	0.634	0.526
*A. platys*			
Age	0.113	1.187	0.235
Sex	-0.069	-0.789	0.430
Lifestyle	0.114	1.445	0.148
Ectoparasitic prevention	-0.003	-0.028	0.978
Breed	-0.151	-1.161	0.246
*Hepatozoon* spp.			
Age	-0.173	-1.966	**0.049**
Sex	-0.054	-0.623	0.534
Lifestyle	0.046	0.399	0.690
Ectoparasitic prevention	0.010	0.079	0.937
Breed	0.007	0.071	0.943
*M. haemocanis*			
Age	-0.127	-1.650	0.099*
Sex	0.030	0.348	0.728
Lifestyle	0.096	0.921	0.357
Ectoparasitic prevention	-0.034	-0.287	0.774
Breed	-0.046	-0.439	0.661
Covariances			
*E. canis* - Leishmaniosis	0.243	2.303	**0.021**
*A. platys* - Leishmaniosis	0.020	0.223	0.824
*Hepatozoon* spp. - Leishmaniosis	0.034	0.393	0.694
*M. haemocanis* - Leishmaniosis	0.099	1.115	0.265
*E. canis* - *A. platys*	-0.031	-0.889	0.374
*E. canis* - *Hepatozoon* spp.	-0.028	-0.312	0.755
*E. canis* - *M. haemocanis*	-0.046	-0.598	0.550
*A. platys* - *Hepatozoon* spp.	-0.098	-1.130	0.258
*A. platys* - *M. haemocanis*	0.072	0.647	0.517
Hepatozoon spp. - *M. haemocanis*	0.158	1.761	0.078*

*Abbreviations*: *A*. *platys*, *Anaplasma platys*; *E*. *canis*, *Ehrlichia canis*; *M*. *haemocanis*, *Mycoplasma haemocanis*

Significant relationships (*P* ≤ 0.05) denoted by bold font and trending relationships (*P* < 0.1) denoted by *

## Discussion

In this first comprehensive case-control study assessing the risk of VBP co-infection in dogs with leishmaniosis, our key finding shows that dogs with ClinL are 12 times (CI: 1.5–106.0, *P* = 0.022) more likely to be co-infected with *E. canis* compared to healthy controls. This further supports the concept of synergism between *L*. *infantum* and *E. canis* during co-infection in dogs in which, as previous studies have suggested, there are more commonly clinical signs (e.g. lymphadenomegaly, splenomegaly, epistaxis, weight loss) [27], more severe haematological changes (e.g. reduced platelet aggregation response, increased activated partial thromboplastin time) [7, 27–29] and hindered clinical improvement during treatment [30] compared to dogs with either ClinL or canine monocytic ehrlichiosis alone.

The pathogenesis behind the speculated synergetic action of *L*. *infantum* and *E. canis* in dogs has not been investigated. Due to the zoonotic nature of canine leishmaniosis there have been extensive studies on the immunopathology of this disease, and it is the best understood canine VBP [9]. It is widely accepted that *L*. *infantum* infection promotes a mixed Type 1 T helper (Th1) and Th2 response that will determine the clinical outcome [31], with increased immunosuppressive substances such as interleukin 10, transforming growth factor β and prostaglandin E2 prevailing in dogs with ClinL [32–35]. The suppression of the immune system by these substances could enable reactivation of a previously subclinical *E*. *canis* infection or facilitate the establishment of a new *E. canis* infection in dogs. While little is known regarding the immunopathology of canine monocytic ehrlichiosis, there is evidence of downregulation of major histocompatibility complex (MHC) class II molecules in a macrophage cell line infected with *E. canis* compared with uninfected macrophages [36]. This downregulation of MHC could impact upon *Leishmania* infection outcome as MHC class II antigen presentation is likely to be an important mechanism in generating an effective cell mediated response to *L. infantum*. Furthermore, MHC Class II genotype has been associated with *Leishmania* specific antibody level and parasite load but not with clinical outcome [37].

In humans there is a well-established synergism between leishmaniasis and human immunodeficiency virus (HIV) [38], with *Leishmania* causing a more rapid progression to AIDS [39] and HIV increasing the risk of developing fatal visceral leishmaniasis [40]. The immunopathology of this synergistic relationship has been documented to arise due to the co-existence of these two pathogens in macrophages, as well as other cells, triggering complex mechanisms involving cellular-signalling and cytokine production [38, 41, 42]. A similar pathogenesis mechanism could potentially exist between *L*. *infantum* and *E. canis* in dogs, since both microorganisms infect monocytes and macrophages. This hypothetical mechanism is supported by the findings of our clinical case-control study in which an association with ClinL was only found with *E. canis* co-infection, but not with *A*. *platys*, *Hepatozoon* spp. or *M*. *haemocanis* that infect predominantly platelets, neutrophils and erythrocytes, respectively [43–45]. Equally, other mechanisms could orchestrate the pathogenesis of the suspected synergistic relationship between *L*. *infantum* and *E. canis* in dogs. Therefore, further studies are needed to investigate how the co-infection of these two pathogens potentially affect the dog’s immune response.

Although, our study is not a cross-sectional epidemiological research project, and the dog population recruited is heavily biased by the inclusion and exclusion criteria, it does provide information for the prevalence of the various VBP tested in the area of Paphos in Cyprus, especially since 65% (92/142) of the samples we collected were from apparently healthy dogs. In the studied population of 142 dogs there is a noticeably high prevalence of *Hepatozoon* spp. (46%), with *H*. *canis* being the only species identified by sequencing, as well as a reasonably high prevalence for *M*. *haemocanis* (23%). Similar prevalences have been reported for *Hepatozoon* spp. and haemoplasmas in the cat population of this island [20], suggesting that the patterns of infection for these two VBP in both the dogs and cats of Cyprus are possibly driven by comparable processes. The prevalence for *E. canis* of 5% (7/142), and for *A*. *platys* of 4% (5/142) in this canine population, are similar to those reported in dogs from other Mediterranean countries [46].

The use of SEM strengthens the findings of our study by confirming the association found between ClinL and *E. canis* and allowed us to simultaneously investigate the effects of demographic, lifestyle and breed on VBP infection, and the associations between the different VBP. Two additional findings were made. The first one was that dogs infected with *Hepatozoon* spp. were more likely to be infected with *M*. *haemocanis* and, to the authors’ knowledge, this is the first time such an association has been reported. This is probably due to the fact that both VBP are suspected to have the same vector *R. sanguineus*, despite their different routes of transmission: host ingestion of the tick for *Hepatozoon* spp. transmission and a tick bite for *M*. *haemocanis* transmission [44, 47]. Secondly, SEM showed that younger dogs were more likely to be infected with each of *Hepatozoon* spp. and *M*. *haemocanis*, which is in agreement with a previous study on dogs infected with canine haemoplasmas from other Mediterranean countries [48] and could suggest that young animals are more intensively exposed to such VBP.

Limitations of our study include selection and observer bias as this is a case-control study, and the geographical restriction of only including one district of Cyprus. Furthermore, the control dogs were recruited on the basis of being clinically healthy, thus they might not be representative of the general canine population. A multicentre prospective longitudinal study design with follow-up monitoring from birth until death would be ideal, but difficult to implement. Even so, the adequate sample size and conclusions which were based on statistical analysis employing different methodologies should allow some generalisation of our findings to other countries with similar environmental conditions and canine VBP prevalence as Paphos, Cyprus. Studies in the future over longer time periods would be beneficial to investigate the possibility of seasonal effects and to determine if the prognosis of leishmaniosis is different when dogs are also co-infected with *E. canis* and other VBPs.

Our finding, that dogs with ClinL are at increased risk of *E. canis* infection compared to healthy dogs, could impact upon the diagnostic and monitoring management of canine leishmaniosis. We recommend that dogs diagnosed with ClinL should be tested for *E. canis* co-infection using PCR on EDTA peripheral blood [49]. Quantitative serological testing can be considered for the diagnosis of active *E. canis* infection but should be interpreted appropriately [46]. Whilst we did not perform any follow up on the dogs with ClinL, to further investigate if there is an ongoing increased risk of co-infections during or after the treatment period, we recommend *E. canis* PCR testing on EDTA peripheral blood if there is clinical or haematological deterioration, such as thrombocytopenia, despite the dog receiving the appropriate anti-*Leishmania* treatment.

If a dog with ClinL is diagnosed with concurrent *E. canis* infection, we recommend simultaneous treatment of both infections. For *E. canis*, the treatment of choice is oral doxycycline at 5 mg/kg twice daily or 10 mg/kg once daily for 4 weeks [46] and for leishmaniosis the appropriate treatment protocol should be based on the clinical stage following The LeishVet Group Guidelines [15]. Furthermore, dogs with ClinL should receive regular and effective protective topical insecticide repellent to prevent infection with *E. canis* by *R. sanguineus* and avoid transmission of *L*. *infantum* to sand flies.

## Conclusions

We showed that dogs with ClinL are 12 times more likely to be co-infected with *E. canis* than clinically healthy dogs in Cyprus. These findings are of a value in the diagnosis and management of leishmaniosis in dogs. We recommend that dogs diagnosed with ClinL should be tested for *E. canis* co-infection using PCR. Further studies should be targeted in investigating the underlying pathology of this association.

## Abbreviations

CI: confidence interval
ClinL: clinical leishmaniosis
CMhp: “*Candidatus* Mycoplasma haematoparvum”
Ct: threshold cycle
ELISA: enzyme-linked immunosorbent assay
EU: ELISA units
HIV: human immunodeficiency virus
MCH: major histocompatibility complex
OR: odds ratio
qPCR: quantitative polymerase chain reaction
SEM: structural equation modelling
VBP: vector-borne pathogen
